# The Chicago Annual Reunion

**Published:** 1885-01

**Authors:** 


### THE CHICAGO ANNUAL REUNION.

The annual banquet of the Chicago Dental Society, upon the 9th of December, was something more than a mere success. Socially it was a triumph, and the visitors from the East and the West went home with their hearts and their stomachs filled to overflowing with Chicago hospitality. We, of the less demonstrative East feel that we have done the fair thing if we receive our guests at our threshold, and make them welcome to all that we have. But that will not serve a western man. He wants to receive them at the city gates with a brass band and fireworks, and would place the whole earth at their disposal. Chicagoans are proud of their city, and they all seem inspired with a determination that every stranger shall be duly impressed with its greatness.

The guests of the Chicago Dental Society were the recipients of unnumbered courtesies. It was "Pop" here and "Prof." there, while "Aurelius" sweetly beamed smiles and attentions on all. In fact, we very much doubt if any one ever saw quite so much "Pop" before, within the same narrow limits.

Dibdin sings :—

> There's a sweet little cherub that sits up aloft,
> To keep watch for the life of poor Jack.

But when the sweet little "Cherub" heads the general conspiracy against the sobriety of staid professional men, it would seem that poor Jack's chances are materially reduced. The annual dinner, though elaborate, was not the chief attraction; the speeches were only so-so, but the conviviality of the occasion was unbounded. At the dinner at the Calumet Club, indeed, social enjoyment ran riot, and there was not one present, with the possible exception of the host of the evening, who did not cast dignity to the winds and set out for a good time. It was an occasion to draw men into closer relations with each other, for the last crust of conventionalism was thoroughly thawed out. To the guests of the Chicago Dental Society the three days spent in that city will remain a petrified memory, fossilized, not as a dead but as an unchanging recollection.

